# Supplementary material for: Infrared thermochromic antenna composite for self-adaptive thermoregulation
Source: Nat Commun. 2024 Oct 22;15:9109. doi: 10.1038/s41467-024-53177-6 (PMC11496700; doi:10.1038/s41467-024-53177-6)
Supplement: Supplementary file 1 — Supplementary Information [file 41467_2024_53177_MOESM1_ESM.pdf]

Supplementary Information

Infrared thermochromic antenna composite for self-adaptive thermoregulation

Francisco V. Ramirez-Cuevas[1,2†], Kargal L. Gurunatha[1,3†], Lingxi Li[1], Usama Zulfiqar[1], Sanjayan Sathasivam[4,5], Manish K. Tiwari[6], Ivan P. Parkin[5], Ioannis Papakonstantinou[1*]

[1]Photonic Innovations Lab, Department of Electronic & Electrical Engineering, University College London; London WC1E 7JE, United Kingdom

[2]Center for Energy Transición (CENTRA), Facultad de Ingeniería y Ciencias, Universidad Adolfo Ibáñez; Santiago 7941169, Chile

[3]Centre for Nano and Material Science (CNMS), JAIN University; Ramanagara Bangalore 562112, India

[4]School of Engineering, London South Bank University; London SE1 0AA, UK

[5]Materials Chemistry Centre, Department of Chemistry, University College London; London WC1H 0AJ, UK

[6]Nanoengineered Systems Laboratory, Department of Mechanical Engineering, University College London; London WC1E 7JE, UK

*Corresponding author. Email: i.papakonstantinou@ucl.ac.uk

† These authors contributed equally to this work

Contents

Supplementary Text....................3
Suplementary Note 1. Characterization of VO_2 antennas....................3
Suplementary Note 2. Emissivity contrast model....................3
Suplementary Note 3. Orientation-averaged scattering and absorption of polydisperse VO_2 rods...4
Suplementary Note 4. T-Matrix mode decomposition....................5
Suplementary Note 5. Analysis of the morphology of the antenna....................6
Supplementary Figures and Tables....................7
References....................16

Supplementary Text

Suplementary Note 1. Characterization of VO_2 antennas

The phase purity of the hydrothermally synthesized and thermally annealed product were analyzed through PXRD. The observed diffraction peaks for the as-synthesized VO_2 rods (

Supplementary Figure **6**a) can be readily assigned to the tetragonal crystalline phase (space group: p4/ncc) of VO_2 (A), which was in very good agreement with literature values (JCPDS card no. 42–0876). Strong intensity peaks of (110), (102), and (220) at 14.80, 25.50, and 29.90^o indicate good crystallinity and high purity of the as-synthesized VO_2 (A) phase. The phase transformation of this sample from VO_2 (A) to VO_2 (M) was carried by annealing the sample under vacuum (0.1 mbar) at 550 ^{o}C and the transformation was confirmed through PXRD peaks (

Supplementary Figure **6**b) which are in very good agreement with the literature values (JCPDS card no.43-1051). Single step hydrothermal synthesis of VO_2 stars sample XRD peaks were also in good agreement with standard monoclinic VO_2 (M) phase.

XPS measurements were carried out on the VO_2 samples to determine the surface composition and oxidation states. Due to their proximity, both the V 2p and the O 1s peaks were collected and fitted together. All data was charge corrected to the lattice oxygen 1s peak at 530.0 eV.[1] V $2p_{3/2}$ peaks were centered at 516.3 and 516.2 for the micro rods and micro stars, respectively matching the V^{4+} oxidation state (Supplementary Figure 7).[1] Secondary peaks associated with V_2O_5 arising from surface oxidation was observed in all the samples. The V $2p_{3/2}$ peaks for V^{5+} were situated at 518.1 eV for the micro rods and at 517.8 eV for the micro stars (Supplementary Table 1).[1] The presence of the V^{5+} spices are attributed to surface oxidation of the samples and has been observed previously in VO_2 powders and thin films.[2]

The thermal characterization of VO_2 (M) rods through DSC shows endothermic phase transition (T_c) at 70.2 °C as represented in Supplementary Figure 8. Similar trend was observed for VO_2 stars.

Suplementary Note 2. Emissivity contrast model

Using Kirchhoff's law,[3] the emissivity of a composite is given by:

$$\epsilon = 1 - \rho^* - \tau^*$$

where ρ^* and τ^* are, respectively, the reflectance and transmittance of the composite.

At small concentration of antennas, the effect of scattering is negligible, and the reflectance of the hot (ρ_h^*) and cold (ρ_c^*) composite can be approximated by the reflectance of the host film without particles, ρ.[4] On the other hand, through Beer-Lamberts law:[4]

$$\tau^* = (1-\rho)e^{-\left(f_v \frac{C_{\text{abs}}}{V_p} + \alpha_0\right)2t_{\text{film}}},$$

where $\alpha_0 = 4\pi\kappa/\lambda$ is the absorption coefficient of the host material, and κ is imaginary part of the refractive index. A factor of 2 is applied in the exponential term to account for the back reflection.

Under these assumptions, we can approximate the emissivity of a composite as:

$$\epsilon = (1-\rho)\left[1 - e^{-\left(f_v \frac{C_{\text{abs}}}{V_p} + \alpha_0\right)2t_{\text{film}}}\right] \tag{S1}$$

Using Eq. (S1) and $\Delta\epsilon = \epsilon_h - \epsilon_c$, we obtain:

$$\Delta\epsilon = \tau_c e^{-2\alpha_0 t_{\text{film}}}\left[1 - \left(\frac{\tau_c}{1-\rho}\right)^{C_{\text{abs},h}/C_{\text{abs},c}-1}\right], \tag{S2}$$

where $\tau_c = (1-\rho)e^{-2t_{\text{fim}}/\Lambda_{\text{abs,c}}}$.

The accuracy of Eq. (S1) against full radiative transfer simulations using Monte Carlo method can be visualized in Supplementary Figure 15. As shown in the figure, the discrepancy becomes significant for particles with large $\langle C_{\text{sca}}\rangle/V_p$.

Suplementary Note 3. Orientation-averaged scattering and absorption of polydisperse VO_2 rods.

First, we computed the orientation-averaged light scattering parameters of a VO_2 rod of width W and length L, $\langle C_{\text{abs}}\rangle_{W,L}$, $\langle C_{\text{sca}}\rangle_{W,L}$ and $\langle \mu_{\text{sca}}\rangle_{W,L}$, in the range $W \in [0.05, 1.4]$ µm and $L \in [1, 50]$ µm. Each rod was simulated at hot and cold phases (Supplementary Figure 13). Afterwards, the effect of size polydispersity was considered by computing the ensembled averaged $\langle C_{\text{abs}}\rangle$, $\langle C_{\text{sca}}\rangle$ and $\langle \mu_{\text{sca}}\rangle$:

$$\langle C_{\text{abs}}\rangle = \sum_{W,L} F_{W,L}\langle C_{\text{abs}}\rangle_{W,L},$$

$$\langle C_{\text{sca}}\rangle = \sum_{W,L} F_{W,L}\langle C_{\text{sca}}\rangle_{W,L},$$

$$\langle \mu_{\text{sca}}\rangle = \frac{1}{\langle C_{\text{sca}}\rangle}\left[\sum_{W,L} F_{W,L}\langle C_{\text{sca}}\rangle_{W,L}\langle \mu_{\text{sca}}\rangle_{W,L}\right],$$

where $F_{W,L}$ is a 2D Gaussian distribution:[5]

$$F_{w,L} = \frac{1}{2\pi\sigma_W\sigma_L\sqrt{1-r_{W,L}}}\exp\left\{-\frac{1}{2\left(1-\rho_{W,L}^2\right)}\left[\left(\frac{W-\bar{W}}{\sigma_W}\right)^2 - 2r_{W,L}\left(\frac{W-\bar{W}}{\sigma_W}\right)\left(\frac{L-\bar{L}}{\sigma_L}\right) + \left(\frac{L-\bar{L}}{\sigma_L}\right)^2\right]\right\},$$

where $\bar{x}$ and σ_x represent, respectively, the mean and standard deviation of a variable x, and $r_{W,L}$ is the correlation coefficient between W and L. Similarly, the ensemble-averaged volume of the sample V_p is given by:

$$V_p = \sum_{W,L} F_{W,L}V_{W,L}$$

where $V_{W,L}$ correspond to the volume of an antenna of width W and length L.

For the results of Figure 1e (main text), $\bar{W}$, σ_W, $\bar{L}$, σ_L and $r_{W,L}$ were obtained from SEM images of the sample (Figure 1c). The gaussian distribution is plotted in Supplementary Figure 10a, the ensemble

averaged $\langle C_{\text{abs}}\rangle$, $\langle C_{\text{sca}}\rangle$ and $\langle \mu_{\text{sca}}\rangle$ in Supplementary Figure 10b, and radiative energy transfer simulations in Supplementary Figure 10c. In the results of Figure 2h (main text), $r_{W,L} = 0$.

Suplementary Note 4. T-Matrix mode decomposition

In the T-matrix method, the scattered field is expressed by a linear expansion into spherical vector wave functions $\vec{M}_{lm}$ and $\vec{N}_{lm}$:[6]

$$\vec{E}_{\text{sca}} = \sum_{\substack{l=1\\ l'=1}}^{\infty} \sum_{\substack{m=-l\\ m'=-l'}}^{l,l'} \begin{bmatrix} T^{MM}_{lm,l'm'} & T^{MN}_{lm,l'm'} \\ T^{NM}_{lm,l'm'} & T^{NN}_{lm,l'm'} \end{bmatrix} \begin{pmatrix} \vec{M}_{l'm'} \\ \vec{N}_{l'm'} \end{pmatrix}$$

where $T^{MM}_{lm,l'm'}$, $T^{MN}_{lm,l'm'}$, $T^{NM}_{lm,l'm'}$ and $T^{NN}_{lm,l'm'}$ are the T-matrix modes. In a more compact form:

$$\vec{E}_{\text{sca}} = \sum_{ij}^{\infty} T_{ij}\vec{\beta}_j$$

where the indexes i or j are defined by the spherical harmonics indexes as:

$$i = 2[l(l+1) + m - 1] + P$$

and $P = 0, 1$ for M and N, respectively. The following table summarizes the corresponding equivalence:

$\boldsymbol{i}$ **(or** $\boldsymbol{j}$**)**	**0**	**1**	**2**	**3**	**4**	**5**	**6**	⋯	**15**	**16**	⋯
l	1	1	1	1	1	1	2		2	3	
m	-1	-1	0	0	1	1	-2	⋯	+2	-3	⋯
P	0	1	0	1	0	1	0		1	0	

The T-matrix elements represent the contribution of electric and magnetic multipoles to the overall electromagnetic response of the structure. For example, the indexes $1, 3$ and 5 ($0, 2$ and 4) represent the of the electric(magnetic) dipole modes in the x, z and y direction, respectively.[7] The T-matrix is diagonal for structures with spherical symmetry,[8] while other off-diagonal T_{ij} terms appear for non-spherical structures.[9]

We performed T-matrix decomposition using the open-source application SCUFF-TMATRIX.[10] The number of modes was chosen using the formula:[11]

$$\langle C_{\text{abs}}\rangle = -\frac{2\pi}{k_0^2}\sum_{ij}\left[\text{Re}(T_{ii})\delta_{ij} + \left|T_{ij}\right|^2\right]. \tag{S2}$$

The equation was used to determine the minimum number of T-matrix modes, where $\langle C_{\text{abs}}\rangle$ was obtained from orientation-averaged scattering simulations.[12] The number of T-matrix modes was determined by analyzing Eq. (S2) in the wavelength range $\lambda \in [8, 13]$ µm (Supplementary Figure 11b)

The results from mode decomposition reveal the mechanism behind the emissivity enhancement of the hot VO_2 antenna (Supplementary Figure 12). For a sphere (ellipsoid with three equal axis) the response is

featured by the contribution of electric and magnetic dipole modes in the x, y and z. Shrinking one of the three ellipsoid axes leads to a disk-shaped particle, whose emissivity is featured by three strong electric dipole modes, one of which dominates the response. The rod shape that results when shrinking two of the three ellipsoid axes reaches the strongest emissivity, with only one strong electric dipole.

Suplementary Note 5. Analysis of the morphology of the antenna

The results shown in Figure 3e-g (main text), are based on a series of steps that include orientation-averaged scattering,[13] Monte Carlo[14] and Beer Lambert (Eq. S1) simulations. In short, for a given structure:

1. We computed $\langle C_{\mathrm{abs}} \rangle$, $\langle C_{\mathrm{sca}} \rangle$ and $\langle \mu_{\mathrm{sca}} \rangle$ (left column Supplementary Figure 15).
2. These results were used as input in Monte Carlo to predict the spectral emissivity of a composite as a function of f_v, such as the results shown in the center column of Supplementary Figure 15.
3. The average emissivity in the atmospheric window was plotted as a function of f_v (right column, Supplementary Figure 15).
4. The value of f_v with the largest $\Delta\epsilon$ was selected and plotted in Figure 3g (main text).

For core@shell and spherical particles, the scattering and absorption cross section was estimated by the scattering theory for multilayered spheres.[15,16]

Supplementary Figures and Tables

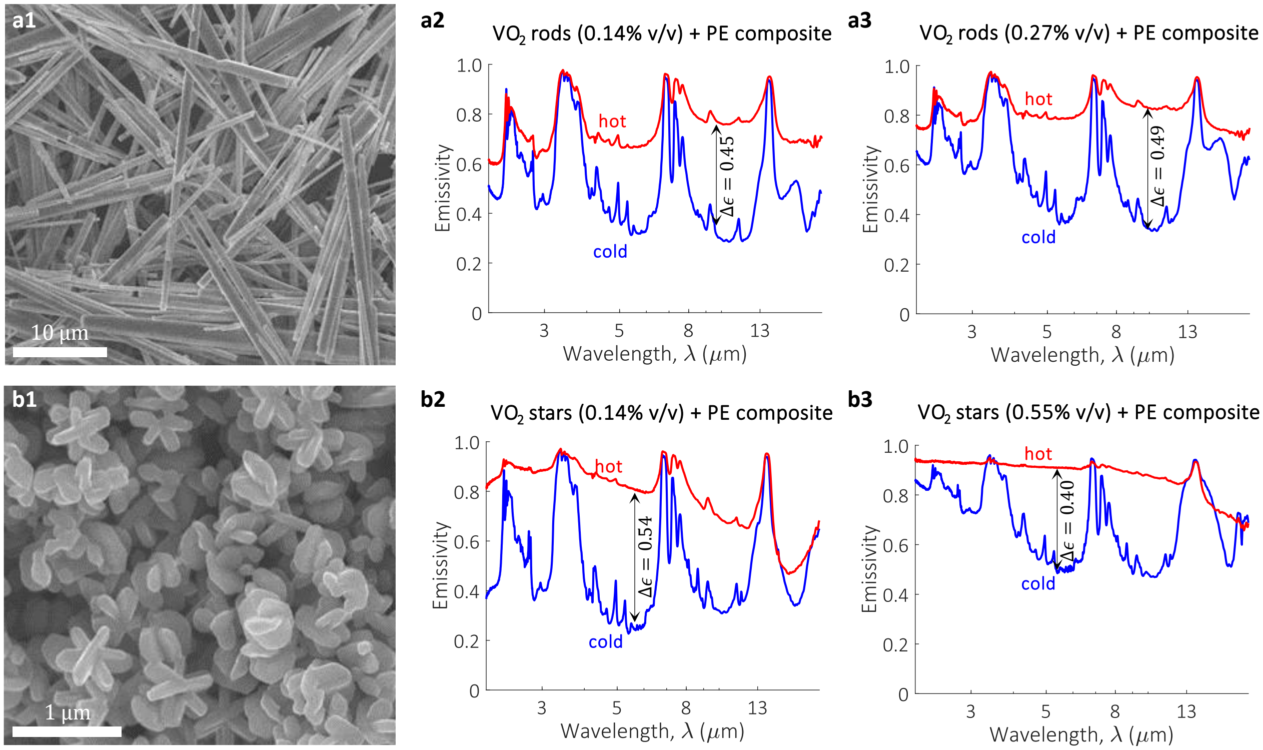

Supplementary Figure 1. a1. and **b1.** SEM images of as-synthesized VO_2 rods and stars used in the results of the main text, respectively. **a2. and a3.** Measured emission spectra of VO_2 rods + PE composites based on the sample shown in a1, and volume fractions of 0.14% and 0.27% v/v, respectively. **b2 and b3.** Measured emission spectra of VO_2 stars + PE composites based on the sample shown in b1, and volume fractions of 0.14% and 0.55% v/v, respectively.

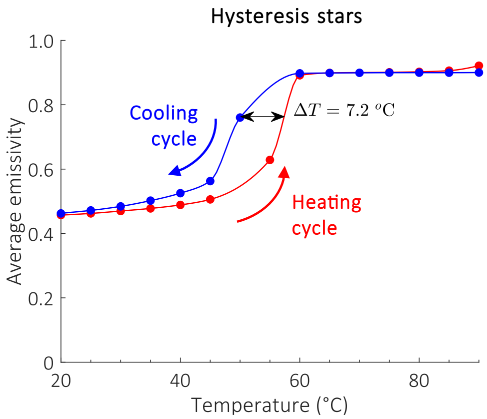

Supplementary Figure 2. Hot/cold phase transition and hysteresis width of the VO_2 stars composite shown in Fig. 3b of the main text. The curves are based on the average emissivity at the range, $\lambda \in 4 - 6$ μm.

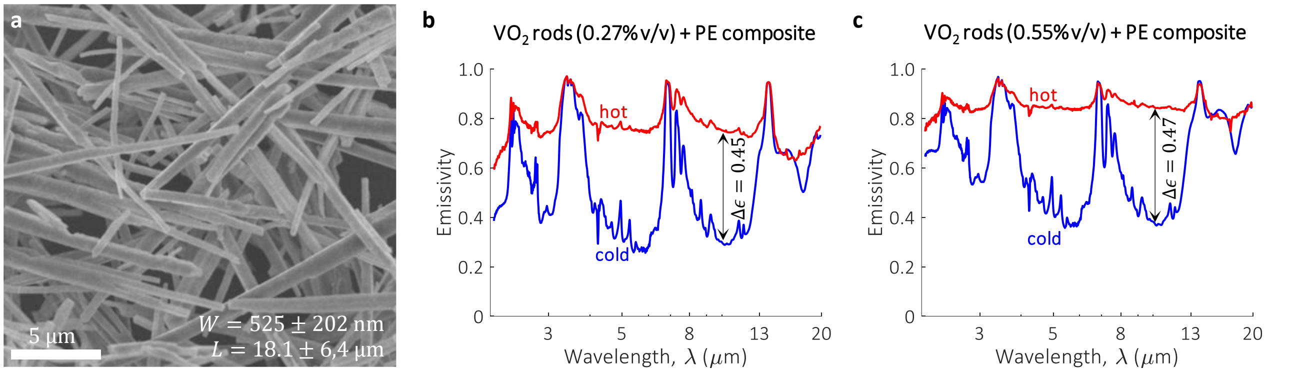

Supplementary Figure 3. a. SEM images of another batch of VO_2 rods, synthesized according to the guidelines outlined in Methods (Sample preparation) **b. and c.** Measured emission spectra of VO_2 rods + PE composites based on the sample shown in a, and volume fractions of 0.27% and 0.55% v/v, respectively.

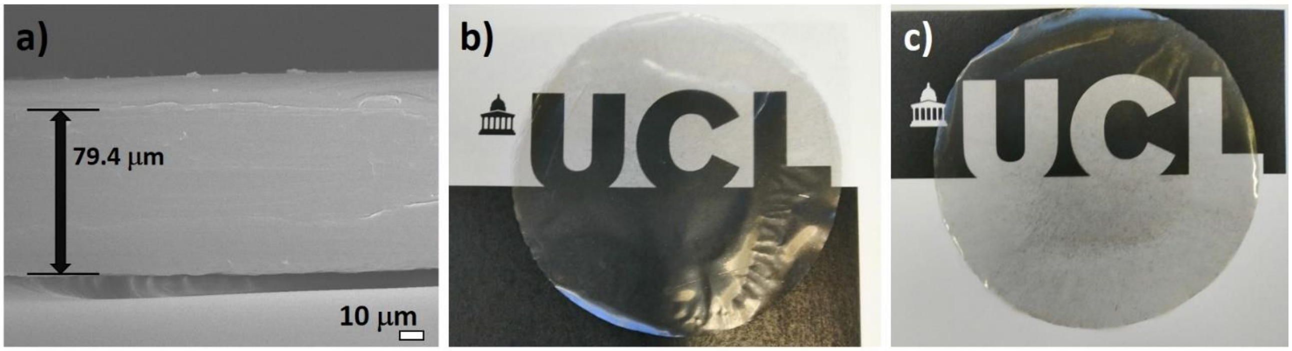

Supplementary Figure 4. a. Cross sectional SEM image of VO_2 polymer composite showing the thickness of 79.4 μm. **b.** & **c.** Compress molded sample of polyethylene and VO_2 rod + polyethylene composite films.

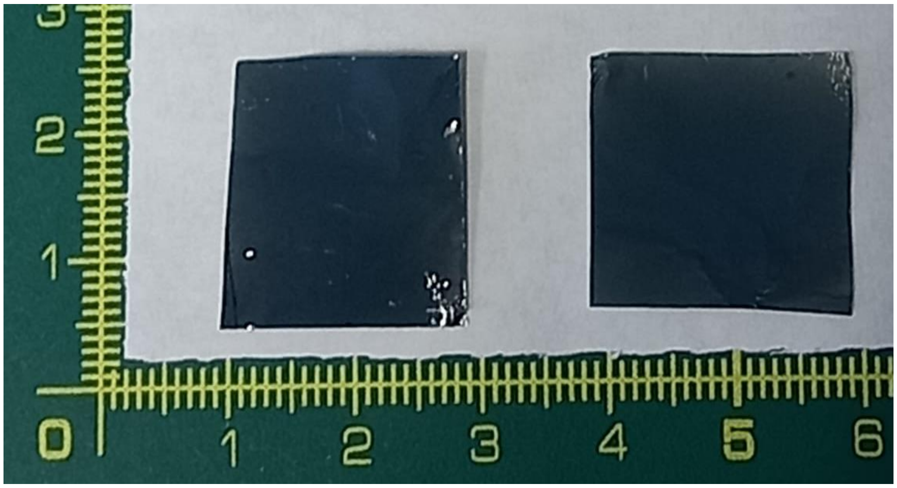

Supplementary Figure 5. Spray coated VO_2 rods on aluminum foil (scale in cm).

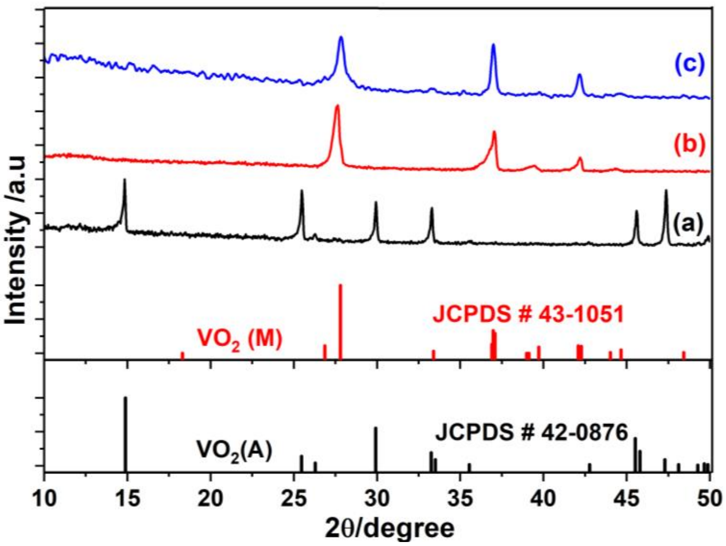

Supplementary Figure 6. Powder X-ray diffraction (PXRD) pattern; JCPDS plots of VO_2 (A) and VO_2 (M), followed by (a) as-synthesized and (b) annealed (550 °C) VO_2 rods, and (c) as-synthesized VO_2 stars.

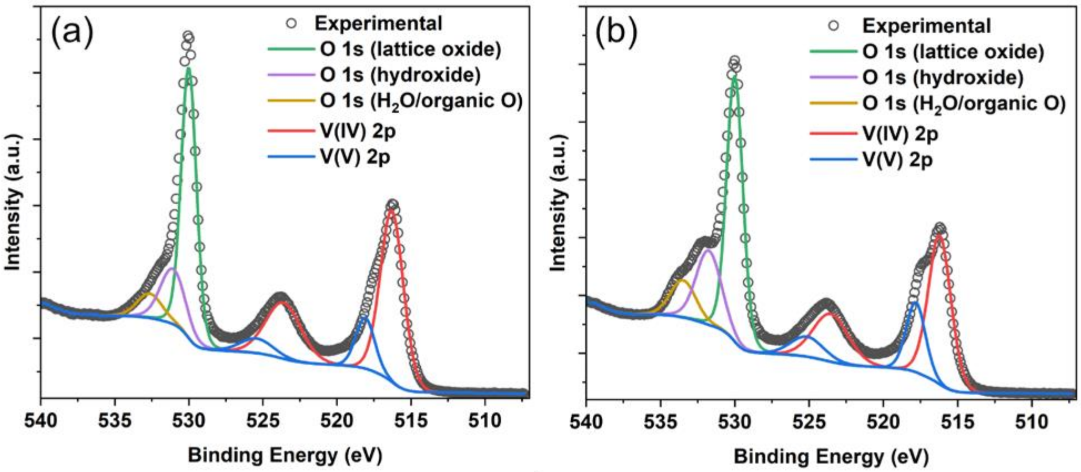

Supplementary Figure 7. High resolution V2p3/2 XPS spectra of a) VO_2 (M) rods and b) stars XPS data showing the O 1s and V 2p experimental (white circles) and peak fitting (colored lines). Only vanadium in the 4+ and 5+ oxidation states was detected in the sample.

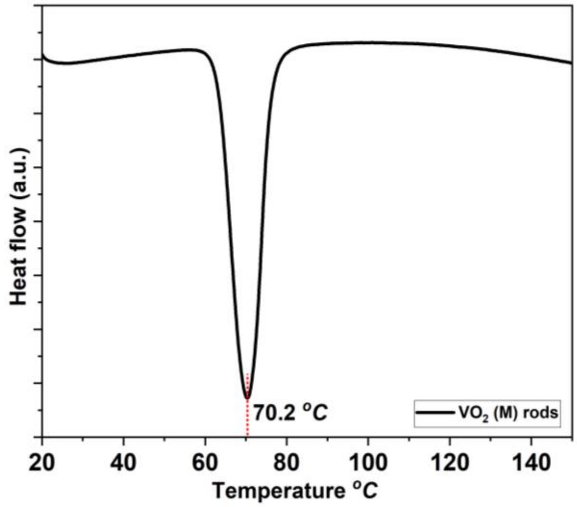

Supplementary Figure 8. Differential Scanning calorimetry (DSC) curve of VO_2 (M) rods showing thermochromic phase transition temperature (T_c) at 70.2 °C.

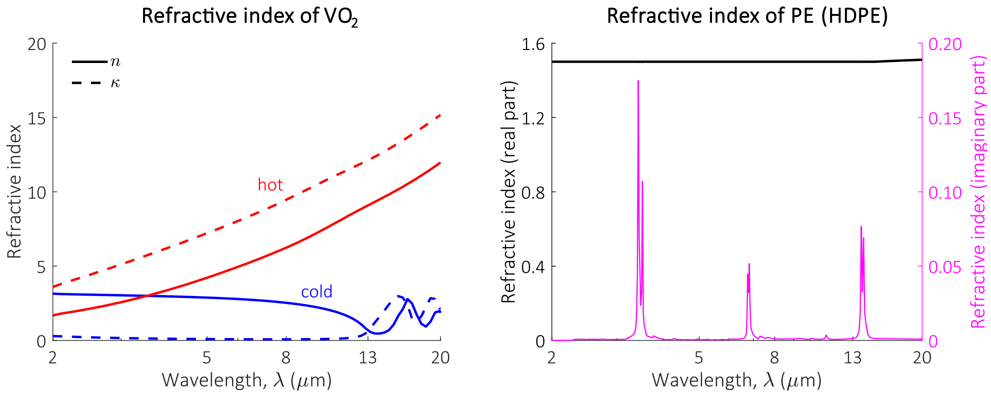

Supplementary Figure 9. Refractive indexes used in all simulations; all curves are reported elsewhere.[17,18] (left) VO_2 at hot/cold phase, based on measurements on sputtered VO_2 films on a sapphire substrate. (right) Polyethylene, which was based on measurements on High-Density polyethylene (HDPE).

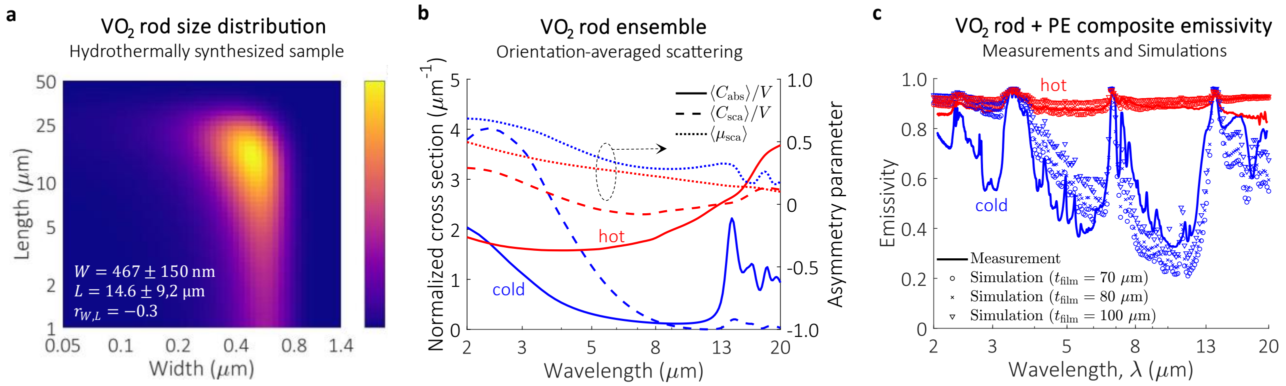

Supplementary Figure 10. a. Size distribution of the sample of VO_2 rod antennas shown in Figure 1c (main text). **b.** Simulated ensemble-averaged $\langle C_{\rm abs}\rangle/V_p$, $\langle C_{\rm sca}\rangle/V_p$ (left vertical axis) and $\langle \mu_{\rm sca}\rangle$ (right vertical axis) of the VO_2 rod antenna ensemble based the size distribution shown in (a). **c.** The results from (b) were used to simulate the emissivity of the composite and compare with the measurements shown in Figure 1e (main text). The simulation considered three film thicknesses ($t_{\rm film}$) within the tolerance measured in the sample (80 ± 15 μm).

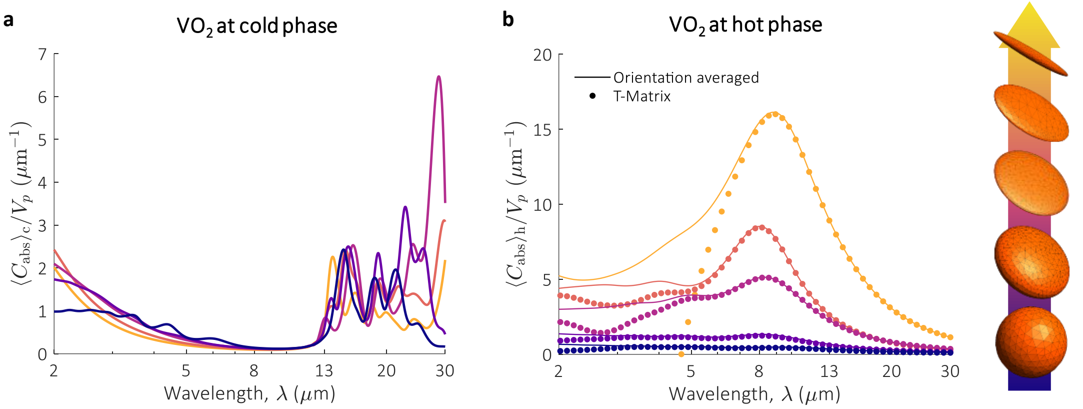

Supplementary Figure 11. Simulated orientation-averaged absorption cross section of a VO_2 ellipsoid at the cold (a) and hot (b) phase, as a function of SA:V. The dimensions are shown in Figure 2c (main text). In figure b, the filled circles represent $\langle C_{\text{abs}} \rangle$ computed by T-matrix mode decomposition, as a method of validation to determine the number of T-matrix modes (see 0).

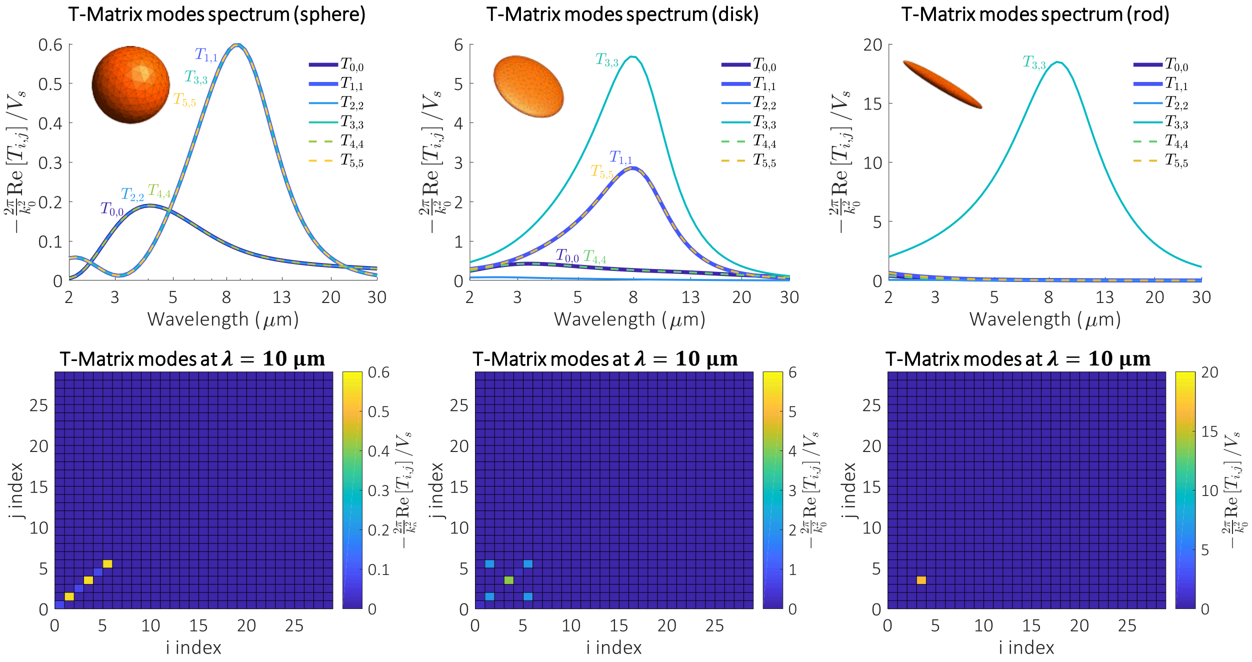

Supplementary Figure 12. T-matrix mode decomposition of a VO_2 particle as a function of its SA:V ratio. Results for a sphere, disk and rod shapes are shown at the left, center, and right columns, respectively. The top row shows the spectral response of the modes at the diagonal of the T-matrix, where $T_{1,1}$, $T_{3,3}$ and $T_{5,5}$ ($T_{0,0}$, $T_{2,2}$ and $T_{4,4}$) represent the electric (magnetic) dipole modes at x, z and y direction, respectively. The bottom row shows the full T-matrix at $\lambda = 10$ μm. In all the figures, the T-matrix modes are expressed as $-\frac{2\pi}{k_0^2}\text{Re}\left(T_{ij}\right)/V_s$ for direct comparison, in accordance with equation (S2).

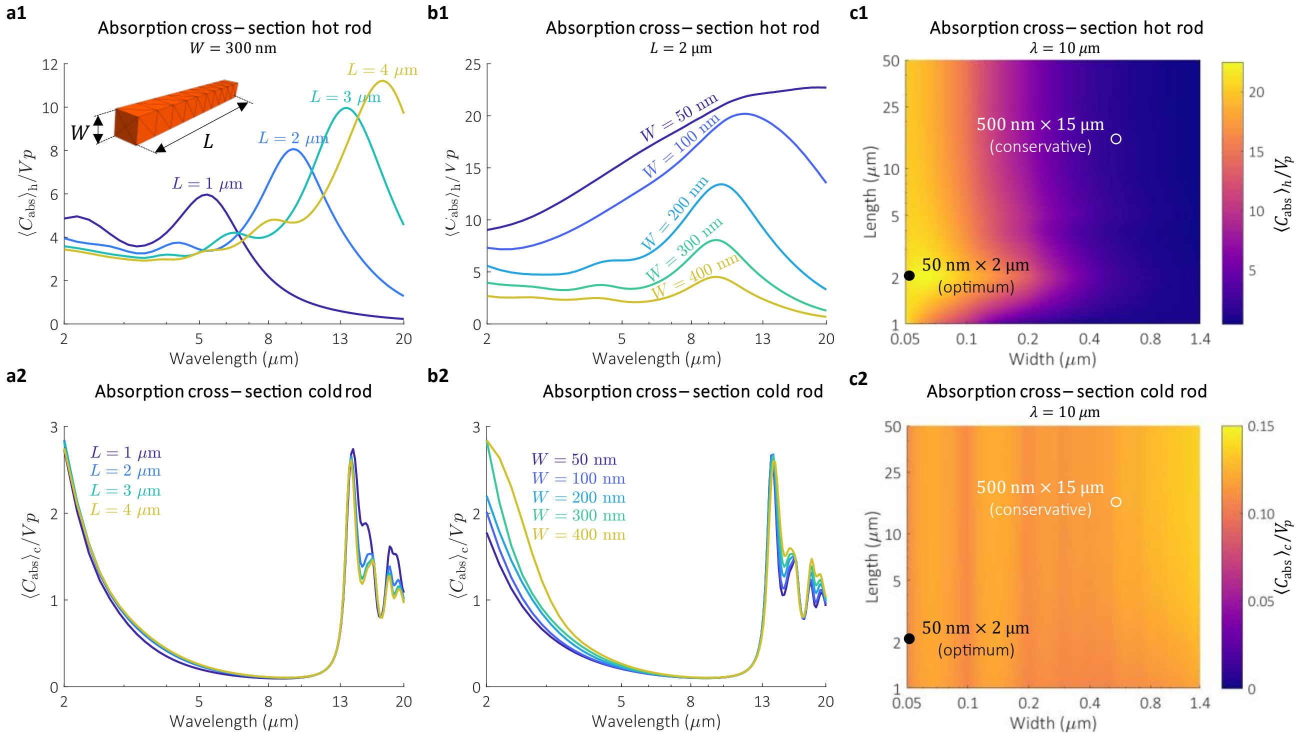

Supplementary Figure 13. a1(a2). and b1(b2). Orientation-averaged absorption cross-section of hot(cold) phase VO_2 rods at as a function of L and W, respectively. **c1(c2)** Orientation-averaged absorption cross-section of hot(cold) phase VO_2 rod at λ=10 μm as a function W and L. The results shown here were used to elaborate Figures 2d-e of the main text.

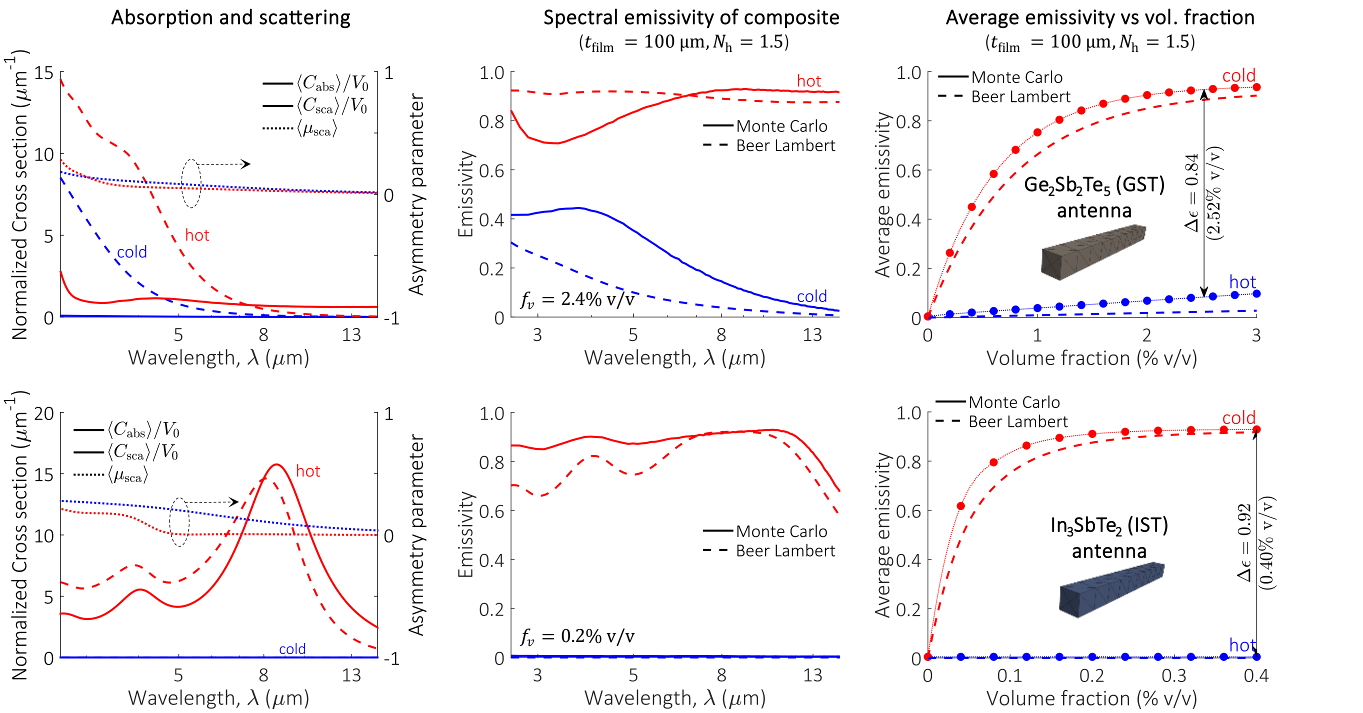

Supplementary Figure 14. Analysis of GST (top row) and IST (bottom row) rod antennas and composites. Each row shows **(left)** normalized scattering and absorption cross section (left vertical axis), and asymmetry parameter (right vertical axis)**;** **(center)** spectral emissivity of a composite based on a transparent host; and **(right)** average emissivity in the atmospheric window of the respective composite against the particle's volume fraction. The refractive index of GST and IST is reported elsewhere.[19,20] In all simulations, the rod width and length is 200 nm and 2 μm, respectively. The results from Beer-Lambert are obtained on Eq. S1 and $\langle C_{\text{abs}} \rangle / V_p$. For the two figures at the right column, each circle corresponds to a Monte Carlo simulation, and the dotted lines are drawn as a guide to the eye**.**

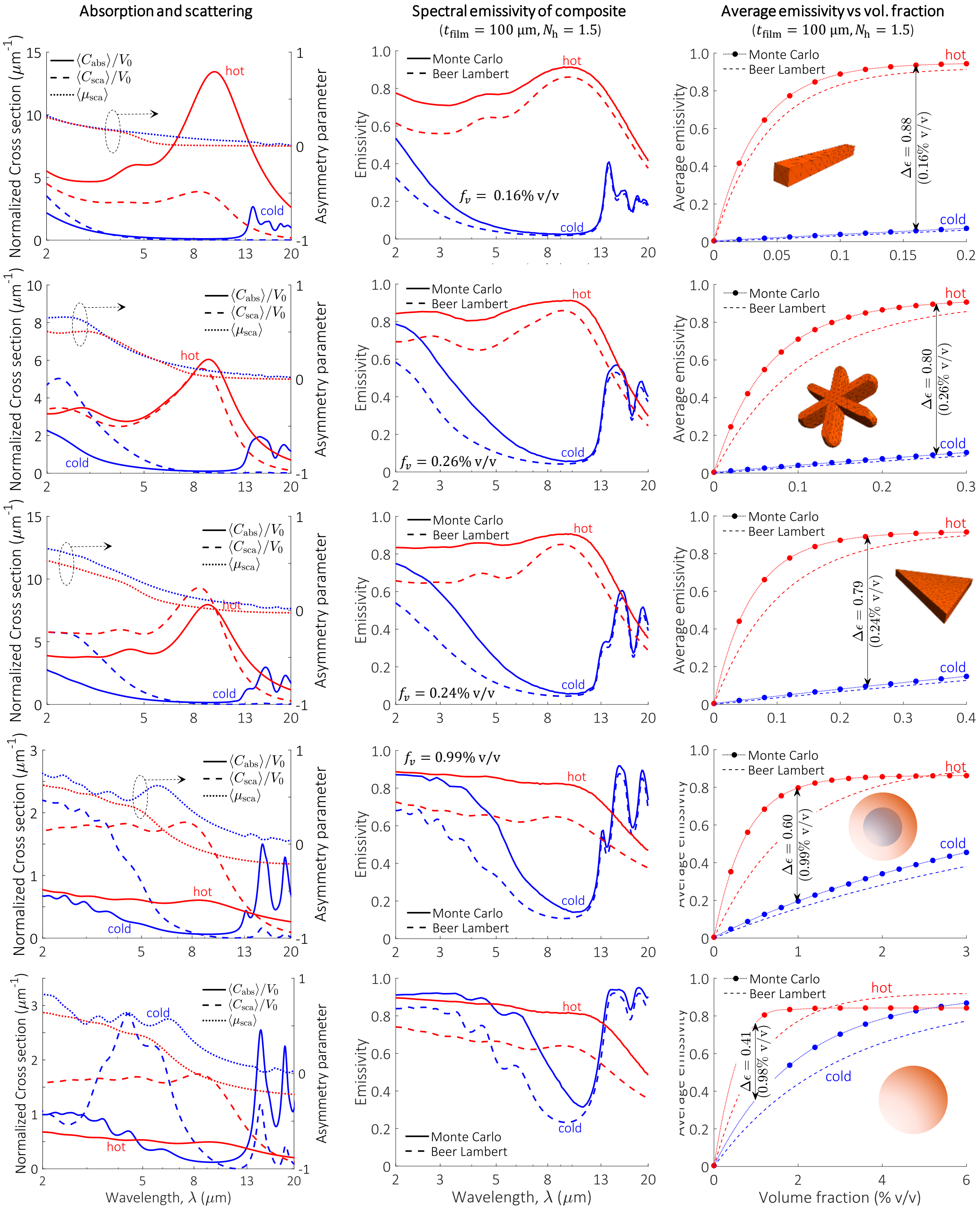

Supplementary Figure 15. (From first row) Simulation of rod, flake and star-shaped VO_2 antennas, core-shell particle with a VO_2 shell VO_2 sphere (at each row), showing **(left)** normalized scattering and absorption cross section (left vertical axis), and asymmetry parameter (right vertical axis)**; (center)** spectral emissivity of a composite based on a transparent host; and **(right)** average emissivity in the atmospheric window of the respective composite against volume fraction. The dimensions of each structure are shown in Fig. 3d (main text). The results from Beer-Lambert are obtained from Eq. S1 and $\langle C_{abs}\rangle/V_p$. For the figures at the right column, each circle corresponds to a Monte Carlo simulation, and the dotted lines are drawn as a guide to the eye**.**

Supplementary Table 1. The XPS results for the VO2 samples grown via hydrothermal methods. Peak positions and V(IV) and V(V) concentrations are given. All peaks were charge corrected to lattice oxygen at 530.0 eV.

	Binding energy / eV		Concentration / at.%	
**Sample**	**V(IV) $2p_{3/2}$**	**V(V) $2p_{3/2}$**	**V(IV)**	**V(V)**
**Micro rods**	516.3	518.1	80.5	19.5
**Micro stars**	516.2	517.8	70.6	29.4

Supplementary Table 2. Mesh refinement of rod, star and flake structures used in the simulation results shown in Figure 3e, 3f and 3g.

**Object**	**Number of triangular panels**		**Absolute error**					
			$\langle C_{\text{abs}} \rangle / V_p$		$\langle C_{\text{sca}} \rangle / V_p$		$\langle \mu_{\text{sca}} \rangle$	
	**Case 1**	**Case 2**	**cold**	**hot**	**cold**	**hot**	**cold**	**hot**
Rod	88	352	3.174e-02	1.745e-02	4.083e-02	1.644e-02	8.322e-04	8.547e-04
Star	1116	1890	2.164e-03	7.249e-03	3.169e-03	6.243e-03	2.414e-04	5.376e-04
Flake	1160	2576	1.303e-03	4.129e-03	1.567e-03	3.956e-03	7.731e-05	2.492e-04

References

1. Biesinger, M. C., Lau, L. W. M., Gerson, A. R. & Smart, R. S. C. Resolving surface chemical states in XPS analysis of first row transition metals, oxides and hydroxides: Sc, Ti, V, Cu and Zn. *Appl. Surf. Sci.* **257**, 887–898 (2010).
2. Powell, M. J. *et al.* Qualitative XANES and XPS Analysis of Substrate Effects in VO2 Thin Films: A Route to Improving Chemical Vapor Deposition Synthetic Methods? *J. Phys. Chem. C* **121**, 20345–20352 (2017).
3. Lavine, A. S., DeWitt, D. P., Bergman, T. L. & Incropera, F. P. *Fundamentals of Heat and Mass Transfer*. (John Wiley and Sons, 2011).
4. Ishimaru, A. *Wave Propagation and Scattering in Random Media*. (Oxford University Press, 1997).
5. Gut, A. *An Intermediate Course in Probability*. (Springer New York, 2009).
6. Tsang, L., Kong, J. A., Ding, K.-H. & Ao, C. O. *Scattering of Electromagnetic Waves: Theories and Applications*. (John Wiley & Sons, Inc., 2000).
7. Poleva, M. *et al.* Multipolar theory of bianisotropic response of meta-atoms. *Phys. Rev. B* **107**, L041304 (2023).
8. Krüger, M., Bimonte, G., Emig, T. & Kardar, M. Trace formulas for nonequilibrium Casimir interactions, heat radiation, and heat transfer for arbitrary objects. *Phys. Rev. B* **86**, 115423 (2012).
9. Mishchenko, M. I., Hovenier, J. W. & Travis, L. D. *Light scattering by nonspherical particles: theory, measurements, and applications*. (Academic Press, 2000).
10. Reid, M. T. H. SCUFF-EM: Free, open-source software for boundary-element analysis of problems in computational physics and engineering. https://homerreid.github.io/scuff-em-documentation/ (2014).
11. Khlebtsov, N. G. Orientational averaging of light-scattering observables in the T-matrix approach. *Appl. Opt.* **31**, 5359 (1992).
12. Ramirez-Cuevas, F. V. AVESCATTER: A SCUFF-EM application for light scatttering of randomly oriented particles of arbitrary shape. https://github.com/PanxoPanza/scattering_random_orientation (2020).
13. Ramirez-Cuevas, F. V., Gurunatha, K. L., Parkin, I. P. & Papakonstantinou, I. Universal theory of light scattering of randomly oriented particles: a fluctuational-electrodynamics approach for light transport modeling in disordered nanostructures. *ACS Photonics* **9**, 672–681 (2022).
14. Ramirez-Cuevas, F. MC-Photon. A monte-carlo software for photon transport. *Github* https://github.com/PanxoPanza/mc-photon.git (2021).
15. Johnson, B. R. Light scattering by a multilayer sphere. *Appl. Opt.* **35**, 3286 (1996).
16. Wu, Z. S. & Wang, Y. P. Electromagnetic scattering for multilayered sphere: Recursive algorithms. *Radio Sci.* **26**, 1393–1401 (1991).
17. Wan, C. *et al.* On the optical properties of thin-film vanadium dioxide from the visible to the far

infrared. *Ann. Phys.* **531**, 1900188 (2019).

18. Palik, E. D. & Ghosh, G. *Handbook of optical constants of solids*. (Academic Press, 1998).

19. Du, K. *et al.* Control over emissivity of zero-static-power thermal emitters based on phase-changing material GST. *Light Sci. Appl.* **6**, e16194–e16194 (2016).

20. Heßler, A. *et al.* In3SbTe2 as a programmable nanophotonics material platform for the infrared. *Nat. Commun.* **12**, 1–10 (2021).
